# Resilience of Health Systems: Understanding Uncertainty Uses, Intersecting Crises and Cross-level Interactions: Comment on "Government Actions and Their Relation to Resilience in Healthcare During the COVID-19 Pandemic in New South Wales, Australia and Ontario, Canada"

**DOI:** 10.34172/ijhpm.2022.7279

**Published:** 2022-06-01

**Authors:** Kayvan Bozorgmehr, Andreas Zick, Tobias Hecker

**Affiliations:** ^1^Department of Population Medicine and Health Services Research, School of Public Health, Bielefeld University, Bielefeld, Germany.; ^2^Institute for Interdisciplinary Research on Conflict & Violence (IKG), Bielefeld University, Bielefeld, Germany.; ^3^Section for Health Equity Studies & Migration, University Hospital Heidelberg, Heidelberg, Germany.; ^4^Faculty of Educational Science, Bielefeld University, Bielefeld, Germany.; ^5^Research Institute Social Cohesion (FGZ/RISC), Bielefeld, Germany.; ^6^Working Unit Clinical Developmental Psychopathology, Faculty for Psychology and Sport Sciences, Bielefeld University, Bielefeld, Germany.

**Keywords:** Resilience, Uncertainty, Governance, Multi-scale Dynamics, Conflict, Health System

## Abstract

The coronavirus disease 2019 (COVID-19) pandemic has created opportunities to study resilience in multiple, interrelated societal systems while considering the institutional, community and individual level. We aim to discuss critical, yet underrepresented, issues in resilience discourses which are fundamental to advance theories, concepts and measurement of health system resilience. These relate to a better understanding of (*i*) how government’s handle and *use uncertainties* to facilitate or impede change, including the role of negotiation and conflicts, (*ii*) the intersections of health with multiple, co-occurring crises (*systemic intersections*), and (*iii*) *cross-level interactions*, ie, the interrelation between individual-level resilience, the collective resilience of groups and communities, and the resilience of a system as a whole (and *vice versa*). Analyses of these aspects can help to "contextualize" our understanding of resilience in complex adaptive systems. However, conceptual clarity is needed whether resilience is considered an underlying feature, outcome, or intermediate determinant of a (health) system’s performance.

 The coronavirus disease 2019 (COVID-19) pandemic has led to a surge of debates on resilient health systems, a notion rooting in ecological sciences^1^ and theories of complex adaptive systems.^2,3^ Resilience refers to a system’s capacity to maintain or restore its functions despite disruptions caused by external factors.^4,5^ The COVID-19 pandemic demands an understanding of resilience and is a unique opportunity to study, and critically advance our knowledge of resilience in multiple, interrelated societal systems while considering the institutional, community and individual level. The pandemic sparked a *whole-society response*, spanning across and linking different societal systems, eg, the health system, the political system, the social and educational system and the economic system. The pre-existing intersections of these societal systems, and their interconnectedness through social behaviour and solidarity,^6^ became pertinently ‘visible’ through the *magnifying function* of the pandemic. The adaptability of “social behaviour in face of existential threat” has barely been demonstrated on such a scale, while the predictability of such behaviour was limited.^6^ Hence, it caused new, and re-enforced pre-existing, intra- and inter-societal conflicts which require collective or institutional, as well as community-based and individual systems of resilience.

 Seizing the moment to rethink health systems, including our understanding of factors that foster resilience,^7^ means that we need to critically advance theories, concepts and measurement approaches of resilience in complex adaptive systems. Empirical studies on health system resilience are rare and skewed towards health care delivery,^4^ so that important domains and health system building blocks, such as governance and related government actions, as well as conflicts in systems (eg, on resources or power) are sidelined. While there is a surge on literature during the COVID-19 pandemic highlighting the relevance of resilience of health systems, only few empirical studies have emerged, and systematic reviews need to take stock of the emerging empirical landscape since the most recent pre-pandemic stock-taking exercise.^4^ Additionally, the different layers of systemic resilience, ie, the structural, group-based or collective, and individual factors determining the system and its resilience, are seldomly differentiated.

 Recent approaches amidst the pandemic tempted, for example, to use “a new health systems resilience framework”^8^ to study resilience factors in health systems, but at the bottom-line fall short in advancing our conceptual understanding and knowledge of system resilience beyond the conventional health system building blocks of the World Health Organization (WHO) published 15 years ago.^9^ Others, such as Smaggus et al,^10^ took a qualitative approach based on a content analysis of official media releases (issued by regional state governments in Australia and Canada), to gain insights into government responses to the COVID-19 pandemic and their relation to health system resilience. Using Hollnagel’s resilience potentials of responding, monitoring, anticipating, and learning^11^ as an overarching framework, they examine how legislative and executive actions of the government, including the communication of such actions, relate to the concept of resilience, how they may contribute to the capacity for resilient performance of the health system, and what opportunities exist to foster resilience in healthcare through government actions. Building upon the study of Smaggus et al, we aim to discuss and touch upon a few critical issues which are yet underrepresented in resilience discourses, but are fundamental to substantially advance theories, concepts and measurement approaches of health system resilience in the future.

## Understanding How Government’s Handle and UseUncertainties

 First, a stronger empirical focus on governance and government action in societies is required, including the political dimension of policy and decision making, as well as studies on power as non-decision making and deliberate inaction. In the field of disaster risk protection, improving governance is considered the single most important priority to reduce vulnerabilities and risks for societies.^12^ The pandemic has shown how the health system response is closely intertwined with political decisions, choices, power,^8,13^ civil and societal solidarity^6^. It further revealed pertinently that our focus on (infra-)structural aspects of systems – which are at the core of preparedness indices like the “Global Health Security Index” – may be misleading when contrasted against country responses and COVID-19 epidemiology and mortality.^14,15^

 Understanding a system’s resilience potentials (to speak with Hollnagel’s^11^ terminology), or its management and resilience capacities (to speak with Blanchet’s^16^ terminology), hence requires a better, ie, a “contextualized,” understanding of how local or national governments and policy-makers understand, handle, cope with, communicate, and react to uncertainties, and how they *make use* of uncertainties to *advance* or *resist* transformative agendas. The potential or capacity to anticipate and handle uncertain events, ie, situations, characterized by a lack of knowledge, not as to cause and effect but rather pertaining to whether a certain event is significant enough to constitute a meaningful cause,^17^ is a core feature of several resilience frameworks.^5,11,16^ As highlighted by Smaggus et al with reference to Hollnagel, this capacity is fundamentally determined by decision-makers’ imagination of different models of the future, and how these different “futures” could unfold.^10,11^ Different modes of mechanistic, probabilistic and realistic anticipation have already been formulated,^11^ but these could be further advanced towards an evidence-informed typology of government actions in their use of uncertainty. In the study of Smaggus et al, the government communication resembled the mechanistic and probabilistic modes of dealing with uncertainties, while their related responses were characterised by an overemphasise on prescriptive measures and protocols to compensate for the incomplete knowledge and uncertainty caused by the pandemic.^10^

 This aspect is interlinked not only with the absorptive or adaptive capacity of a system, but especially with its, empirically widely neglected^4^, transformative capacity. Keeping in mind the unpredictability of social behaviour under crises situations,^6^ we need to advance our understanding of why, how, and to which end uncertainties are *used* by decision-makers to advance or impede change, and how these *uses* shape the response to existential threats such as the pandemic. For example, evidence is accumulating that COVID-19 related excess mortality is higher among countries with populist governments, which adopted inadequate and opportunistic policy responses to the pandemic.^18^ Furthermore, addressing the uncertainties by the pandemic through hope-oriented instead of fear-oriented government communication came along with higher adherence and acceptance of control measures.^19^ Understanding of the variation in country response to COVID-19 is slowly growing,^20^ but much more research on the decision-making process in light of existential uncertainties is needed to understand why country responses varied, and why some performed better than others despite having the same or comparable pre-conditions based on their health system characteristics.^8^ The analysis of government communications is a good starting point to approximate government actions,^10^ but rhetoric and practice may diverge. Further research is required on the practice and implementation of actions in situations of uncertainty, including decision-making and negotiation processes, as well as conflicts arising from such actions.

## Intersecting Crises, Conflicts and Cross-level Interactions

 It is acknowledged that resilience capacities need to consider the way how multi-scale dynamics are handled^16^ and that the concept of health system resilience must expand beyond medicine to include social, economic and political factors in society.^8^ However, intersections of the health system with other systems, or with multiple, co-occurring crises are yet neither conceptually^5^ nor empirically^4^ taken into account. For example, the COVID-19 pandemic in Europe has intersected with the 2008-financial crises, large-scale refugee migration in 2015,^21-23^ and more recently with war in Ukraine. The intersections between geopolitics, inter- and intra-societal conflict, and large-scale forced migration^24^ re-enforce each other, but are barely considered when conceptualising or studying system resilience, let alone when designing policy responses. In the study of Smaggus et al, the COVID-19 pandemic intersected with climate-change related challenges, eg, droughts, bush-fires, and floods^10^ in the form of a linked social-ecological system, in which vulnerabilities affect its resilience.^25^ Beyond the domain of learning, few references are made in the literature to the question of how such intersecting and co-occurring crises relate to a system’s resilience potentials and how the study of commonalities and differences in responses to multiple systemic crises can inform our knowledge on resilience.

 This is particularly relevant for transformative resilience, ie, changes beyond immediate absorption or short-term adaptations. For example, COVID-19 pandemic control policies, and the dominating health lens in all areas of life, have created tensions, conflicts, and led to overt resistance as well as blatant violence against health workers,^26^ the health system, and representatives and institutions representing liberal democracies.^27^ The pandemic has not only accelerated knowledge production and health system responses (eg, creating vaccines in ‘worb speed’), but also boosted radicalisation, polarised societies, and catalysed resistance against ‘the system’. What can be observed here, we postulate, can only be understood by studying the systemic interactions, intersections and interrelations of multiple societal systems, including the individuals, agencies, and actors that form and shape them. Understanding the underlying dynamics of such conflicts during uncertainties or transformation requires a focus on the role of politically polarized groups, individuals and institutions, and a particular attention to ideologies, ideas, and interests of actors that may collide within and across systems.

 Ultimately, the underlying societal conflicts will need collective or institutional,^6^ individual and group-based systems of resilience if we expect them to be managed in a constructive (and not disruptive) way. The current models of health system resilience^5^ are, however, agnostic to the these interactions at different levels. It is yet not considered how individual-level resilience factors affect the collective resilience of groups and communities and the resilience of a system at the macro-level (and *vice versa*). In complex adaptive systems, the performance and adaptability of such systems is a function of the actions and decisions taken by the individuals and groups.^2^ This is – for example – reflected in concepts of ‘every day resilience,’^28^ or in situations where, eg, the individual psychological resilience to deal with uncertainties or existential threats affects collective resilience (of groups or communities)^6^ or functions of institutions. Different ‘units’ of resilience emerge also in the media communication analysed by Smaggus et al, ranging from the “resiliency of everyday people” and “extraordinary resilience” of communities to “pandemic-resilient infrastructure” and “whole-of-government” efforts. However, we still lack a clear understanding – both conceptually and empirically – how individual-level resilience, group-based or community-resilience, institutional resilience, and a system’s resilience - corresponding to nested systems ranging from micro to macro dimensions^29^ - interact with each other. The pandemic sparked a *whole-society* response and had impacts on multiple levels. It is hence a perfect opportunity to study such “cross-level interactions.” Analyses which conceptually and empirically span and link the individual, organisational, and societal resilience capacities may help to better contextualize our knowledge on system resilience.

## Conceptual and Analytical Clarity as Pre-condition

 To advance our understanding and knowledge of such ‘contextualized’ system resilience through analyses of uncertainty uses, systemic intersections, or cross-level interactions, we need conceptual and analytical clarity. In view of the empirical landscape on health system resilience^4^ and the non-exhaustive view of the emerging literature,^5,8,10^ we argue that agreement (or at least clarity) is needed whether resilience is studied as outcome, mediator, or determinant of a system’s performance.

 Some studies use these interchangeably: resilience as underlying feature or potential, required to achieve a given outcome, while at the same time the system “was” or “proved” resilient. This creates confusion as it is theoretically and conceptually different whether “resilience” is a *latent* feature of a system (ie, not directly observable), affecting the performance of - let’s say - the system’s observable elements and their measurable performance, or whether “resilience” is an observable *outcome*, defined as a *function of* or *determined by* the performance of the system’s building blocks.^8^ If it is conceived an outcome, that is affected by its own latent feature, models and approaches need to capture this reciprocal relation and the feedback loops (and the resulting ‘endogeneity’). This understanding would also differ from meta-frameworks which define resilience potentials or capacities as *intermediate* factors on the pathway from health system building blocks to its goals (measured by access, quality, safety, and coverage).^5^ We do not seek to conclude on rights or wrongs, but we dare to say that more conceptual and analytical clarity is needed during the endeavor and journey to advanced theories, concepts and measurements on resilience in complex adaptive systems.

## Ethical issues

 Not applicable.

## Competing interests

 Authors declare that they have no competing interests.

## Authors’ contributions

 Conception: KB. First and final draft: KB. Critical revision for important intellectual content: AZ, TH.

## Funding

 This work was supported by the German Science Foundation in the scope of the NEXUS study [DFG grant no: GZ: BO 5233/1-1 – FOR 2928; grant holder: KB] as part of the PH-LENS Research Unit.
